# Formation of Nanoporous Anodic Alumina by Anodization of Aluminum Films on Glass Substrates

**DOI:** 10.1186/s11671-016-1412-y

**Published:** 2016-04-16

**Authors:** Tetyana Lebyedyeva, Serhii Kryvyi, Petro Lytvyn, Mykola Skoryk, Pavlo Shpylovyy

**Affiliations:** V.M. Glushkov Institute of Cybernetics, National Academy of Sciences of Ukraine, Glushkova Ave., 40, Kyiv, 03680 Ukraine; V.E. Lashkaryov Institute of Semiconductor Physics, National Academy of Sciences of Ukraine, pr. Nauki, 41, Kyiv, 03028 Ukraine; NanoMedTech LLC, Antonovycha Str., 68, Kyiv, 03680 Ukraine

**Keywords:** Aluminum thin films, Magnetron sputtering, Thin film anodization, Porous anodic alumina, Metal-clad waveguide, 82.45.Cc, 81.15.Cd, 68.37.-d

## Abstract

Our research was aimed at the study of aluminum films and porous anodic alumina (PAA) films in thin-film РАА/Al structures for optical sensors, based on metal-clad waveguides (MCWG). The results of the scanning electron microscopy (SEM) and atomic force microscopy (AFM) studies of the structure of Al films, deposited by DC magnetron sputtering, and of PAA films, formed on them, are presented in this work.

The study showed that the structure of the Al films is defined by the deposition rate of aluminum and the thickness of the film. We saw that under anodization in 0.3 M aqueous oxalic acid solution at a voltage of 40 V, the PAA film with a disordered array of pores was formed on aluminum films 200–600 nm thick, which were deposited on glass substrates with an ultra-thin adhesive Nb layer. The research revealed the formation of two differently sized types of pores. The first type of pores is formed on the grain boundaries of aluminum film, and the pores are directed perpendicularly to the surface of aluminum. The second type of pores is formed directly on the grains of aluminum. They are directed perpendicularly to the grain plains. There is a clear tendency to self-ordering in this type of pores.

## Background

The researches on the formation of porous anodic alumina (PAA) films for the use of their nanoscale properties can be divided into two main areas: growing oxide on a bulk aluminum, foils, and plates and on films deposited on the substrate. The development of technological processes of PAA creation on bulk aluminum usually presumes the improvement of methods of ordered structures creation with implementation of a two-stage process of anodizing. This is where significant success has already been achieved [1]. However, further use of the PAA films requires their separation from aluminum substrate and transfer and fixation on other carrier. Considering the high fragility of the PAA films, such processes are difficult to perform and are non-technological. Nanostructured PAA films growth on thin Al layers deposited on hard and flexible substrates, which are already widely used in microelectronics, seems to bring more promising results. This allows to combine microelectronic processes with the process of growing of nanostructured PAA layer. However, the method of growing of self-ordered PAA by two-stage anodizing on thin aluminum films (thickness of less then 1000 nm) is not applicable. Only the processes of nanopores ordering for PAA on thin Al films by pre-texturing by special stamps or by ion beam etching through an electron beam lithography fabricated mask can be considered as those that secured proved results [2–9].

To date, there are several directions in which the work on creation of PAA film-based combined nanostructures is being carried out—magnetic nanomaterials, photoelectrochemistry [3], creation of Si nanoholes [4], and valve-metal oxide nanodots or nanorods [5–7]. One of the rapidly developing areas, where PAA films are applied, is the ultra-thin mask of anodic alumina (UTAMs) for the production of ordered nanostructures on substrates [8]. Current works on the formation of arrays of various kinds of nanoelements—nanodots, nanowires, nanorings, etc.—are quite successful.

We can consider as a separate area of research among the mentioned above the creation of nanostructured anodic alumina of optical sensors [10]. The nanostructured surface of the PAA of these sensors can itself have both a functional purpose—that is, to be part of a sensor, such as a waveguide one [11, 12], and serve as a matrix for growing of the elements that interact with the radiation input [13].

For some optical sensors, it is necessary to form PAA film on a transparent substrate. There are many reports on studying the formation of PAA from deposited Al films on glass [11, 14–19]. Analysis of published data shows that during the formation of PAA from aluminum film on any substrate, not only the process of anodic oxidation affects the outcome but also the way of deposition of aluminum films [20], the impurity in the film [21], the heat treatment of aluminum film [22], the thickness of Al film [23], and the presence of a conductive underlayer on an insulating substrate [17, 18].

A comprehensive description of the formation of anodic porous alumina films (as opposed to PAA on a massive aluminum) has not been created so far. Therefore, the results to form PAA received on specific aluminum films and study the influence of the characteristics of aluminum films on the characteristics of PAA constitute great interest.

The aim of our work is to study the formation of PAA from aluminum films, prepared by the method of DC magnetron sputtering at different deposition parameters. We studied the films with a thickness of 200–500 nm. This is exactly the type of PAA films used by us for the waveguide layer of the optical sensors, based on metal-clad waveguides (MCWG) [12]. The particularity of our study is that the investigations were carried out on freshly deposited aluminum films, whose structure has not been subjected to changes by annealing, aging, or chemical surface treatment.

## Methods

### Deposition of Aluminum Thin Films

Optically polished flint glass plates with *n*_D_ = 1.609, a size of 20 × 25 mm, and a thickness of 1 mm were used as substrates. The glass had been pre-cleaned in a chromic acid solution for 6 h and after washing in deionized water was purified in an RF oxygen plasma. Immediately before deposition of metal films, the substrates were being subjected to final cleaning in a way of bombardment of their surface with argon ions with an energy of 400 eV for 10 min in a vacuum chamber. 

Parameters related to the deposition of aluminum films by DC magnetron sputtering are described bellow.

Target, aluminum (99.999 % purity)

Gas, argon (99.95 % purity)

Base pressure, 1 × 10^−6^ Torr

Argon pressure, 1.8 × 10^−3^ Torr

Substrate-to-target distance, 45 mm

The principles of the four series of experiments are described in Table 1.

**Table 1:** Characteristics of the sputtered aluminum thin films

Sputtering parameter of Al	1st group, samples			2nd group, samples		3rd group, samples			4th group, samples		
	1	2	3	4	5	6	7	8	9	10	11
Deposition rate (nm/s)	0.55	1.1	2.2	0.55	0.55	1.1	1.1	1.1	0.55	1.1	2.2
Film thickness (nm) (±5 %)	340	340	340	230	340	230	340	510	230	230	230

### Investigation of Al and PAA Films by SEM and AFM

The surface of the aluminum films was studied by scanning electron microscopy (SEM) and atomic force microscopy (AFM) techniques. For SEM studies of the surface of aluminum samples and PAA, we used Tescan Mira 3 LMU (Tescan Orsay Holding) at 10 kV. A niobium layer with a 10-nm thickness was used as the coating that prevents charging of PAA dielectric surfaces of the specimen.

Scanning probe microscope IIIa NanoScope Dimension 3000 (Digital Instruments/Bruker, USA) was used for AFM. The measurements were performed in a tapping mode with silicon probes with a nominal tip radius of 10 nm.

### X-ray Diffraction

X-ray diffraction (XRD) in our study was carried out using X’Pert Pro MRD diffractometer (PANalytical B.V., Almelo, The Netherlands) with Cu_Kα_ radiation (*λ* = 0.15418 nm) in a grazing geometry (the angle of incident beam was *ω* = 1°). XRD data were collected in the range of 2θ = 35–85° at room temperature. Measurements were performed with scanning step 0.02°, and the time set at each position is 1 s. The tube voltage was 45 kV, and the current was 40 mA. The phase analysis was performed using a database ICDD, PDF-2 Release 2012 by program Crystallographica Search-Match Version 3, 1, 0, 0.

### Anodization

Area of anodizing (approximately 250 mm^2^) was limited by the photoresist mask. The photoresist mask was coated by the stamp to prevent etching of freshly deposited aluminum surface by alkaline developer. Drying of the photoresist was being performed at 100 °C for 20 min.

Anodic oxidation to form PAA films was performed with involvement of a specially created home-made computerized power source. The software provided management of modes of the source, maintained the accuracy of the specified modes of anodizing within 1 %, and allowed recording of the time dependence of *I*(*t*), *V*(*t*), dI/dt(*t*), and dV/dt(*t*) in the anodizing process. Anodic oxidation of aluminum films was held in a two-electrode cell. Vacuum deposited gold film on a glass substrate served as a cathode. PAA formation was carried out in the potentiometric mode under 40 V, and the output of a predetermined voltage was set at a constant current density of at least 50 mA/mm^2^. 0.3 M solution of oxalic acid was used as the electrolyte. Temperature of electrolyte was 13 °C. After the experiments, the samples were rinsed with deionized water and subsequently dried in air.

## Results and Discussion

### Study of the Aluminum Films Surface by SEM Technique

**Fig. 1:**
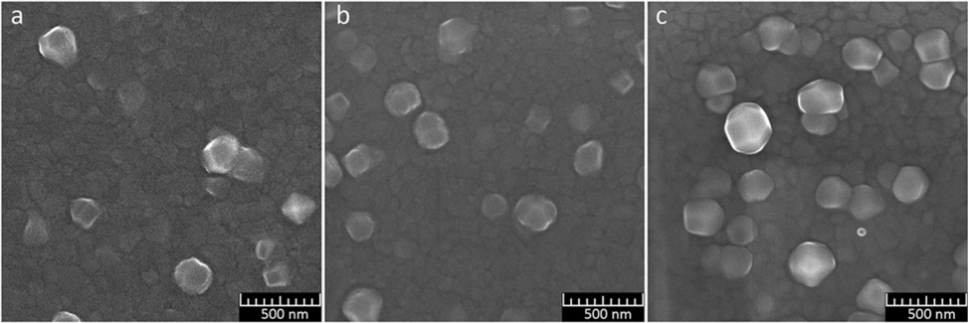
SEM images of the surface of aluminum films of the 1st group of samples. **a** Sample 1. **b** Sample 2. **c** Sample 3

The results of the research of films surface with a thickness of 340 nm formed under different deposition rates—the 1st group of samples—are shown in Fig. 1.

We believe that the structure of our aluminum films can be conveniently described as a combination of a base film with a small size of grains and low roughness and large grains (crystallites), protruding above its surface. SEM images show that the grains of all films greatly vary in their sizes. Size and number of crystallites protruding above the surface increase with higher deposition rate of aluminum. Protruding grains have distinct faces.

Unfortunately, there is no way to determine correctly the average grain size by our images (samples of the 1st, 2nd, and 3rd groups) for their quantitative comparison. The low contrast of the images makes their processing by usual software impossible. However, it is visually noticeable that the increase of the rate of deposition and the thickness of the film leads to the increase in the average grain size of the base and in protruding crystallites.

We carried out the estimation of ranges of protruding grain sizes by processing in the manual mode of SEM images of the samples of the 1st, 2nd, and 3rd groups, using the SEM software.

The ranges of protruding grain size for Al films with a thickness of 340 nm (1st group) at different deposition rates estimated from SEM images are 80–220 nm, 90–280 nm, and 120–310 nm for deposition rates 0.55, 1.1, and 2.2 nm/s, accordingly.

**Fig. 2:**
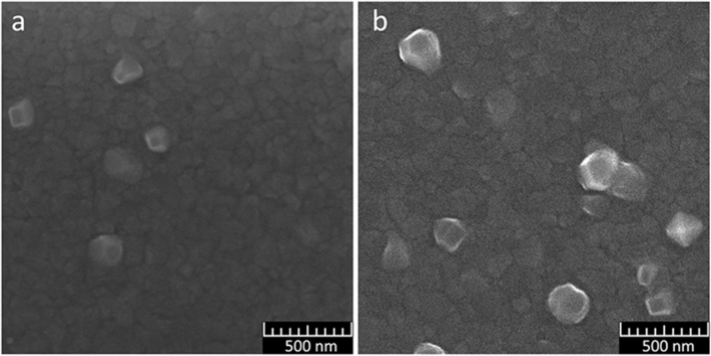
SEM images of the surface of aluminum films of the 2nd group of samples. **a** Sample 4. **b** Sample 5

The SEM results for films of different thickness (2nd group of samples), formed at a current of *I* = 0.125 A, which is the lowest possible for our sputtering system at a given pressure, are shown in Fig. 2. The deposition rate in this manner is 0.55 nm/s. The SEM results show an increase in the average grain size of the base and crystallites with an increase of the film thickness. On some larger crystallites, we can see stratification of their structure (Fig. 2b), analogous to the «ridges» described in researches [24, 25]. Ranges of protruding grain size for Al films of the 2nd group deposited at a deposition rate of 0.55 nm/s were estimated from SEM images. They are 80–220 nm and 150–250 nm for thickness 230 and 340 nm, accordingly.

**Fig. 3:**
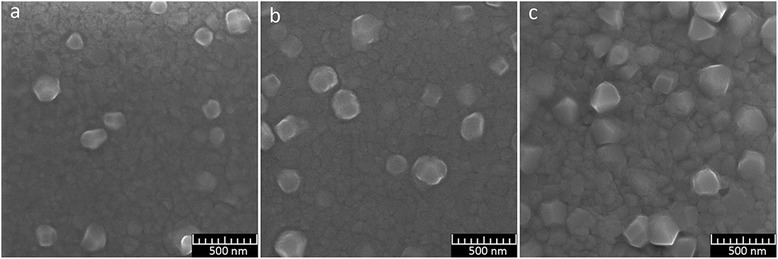
SEM images of the aluminum films surface of the 3rd group of samples. **a** Sample 6. **b** Sample 7. **c** Sample 8

The SEM results for films of different thickness, deposited at a rate of 1.1 nm/s (3rd group of samples) are shown in Fig. 3. The SEM images show that in all films of the 3rd group, grain sizes of the base differ greatly. There is a marked increase in size and number of protruding crystallites, where film thickness was increased. Comparison of films of the same thickness (samples 5 and 7), deposited at different rates, shows that the films, deposited at higher speed, also have a greater number of protruding crystallites. Ranges of protruding grain size for Al films of the 3rd group were estimated for films with thickness of 230, 340, and 510 nm as 110–250 nm, 150–250 nm, and 150–300 nm, accordingly.

### The Study of Aluminum Films by AFM

**Fig. 4:**
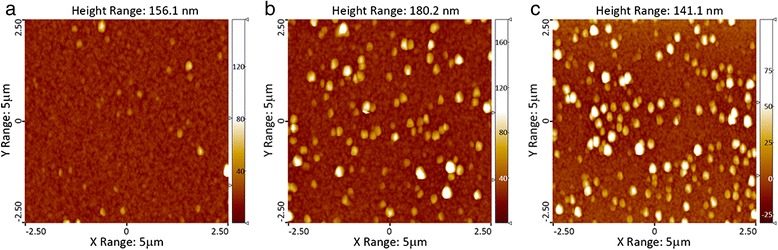
2D image of the surface of the aluminum films of 4th group of samples. **a** Sample 9. **b** Sample 10. **c** Sample 11

The AFM research was performed to receive more information about the surface topography of aluminum films. For this purpose, we explored the surface of the aluminum films with a thickness of 230 nm, deposited at different speeds (4th group of samples). The AFM results are shown in Fig. 4.

These data are in complete agreement with those which were received by the SEM technique. In Fig. 4, we can see that all three aluminum films consist of a base with smaller crystals and also of a number of grains of aluminum, which are protruding above the surface of the substrate. AFM data confirm that the increased power of the magnetron (increase in the deposition rate) results in the increase of the size and the number of grains of aluminum, protruding above the surface of the substrate.

**Fig. 5:**
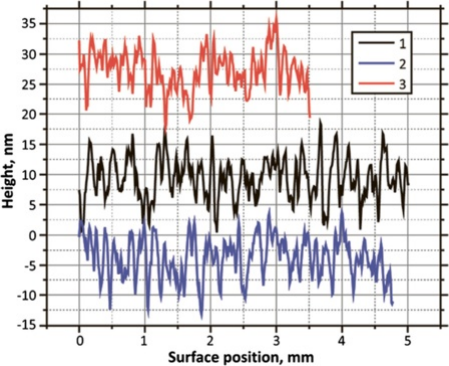
Height profiles of the surface of samples 9, 10, and 11 between the crystallites

The analysis of base surface relief (Fig. 5) also showed an increase in average grain size in response to the gradual increase of discharge power. However, the roughness of substrate surface (RMS) changed only slightly and, for all three films, is about 3 nm.

**Fig. 6:**
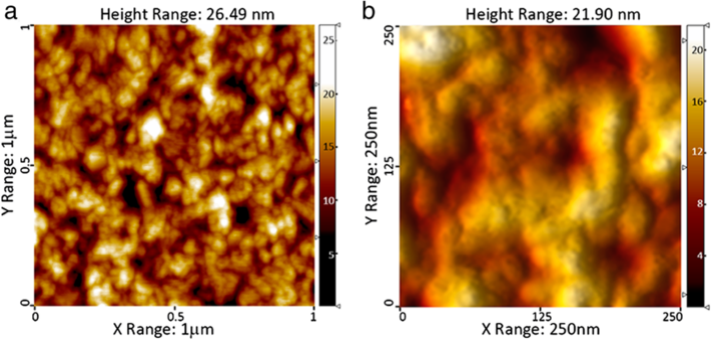
2D AFM images of a homogeneous area between crystallites (sample 9). **a** A field of 1 × 1 μm. **b** A field of 250 × 250 nm

Due to the AFM data, this area can be described as closely packed grains, 50–200 nm in size with a clear subgrain structure (Fig. 6). The typical size of the substructure of grains in the samples of the 4th group lies within the range of 15–30 nm.

**Fig. 7:**
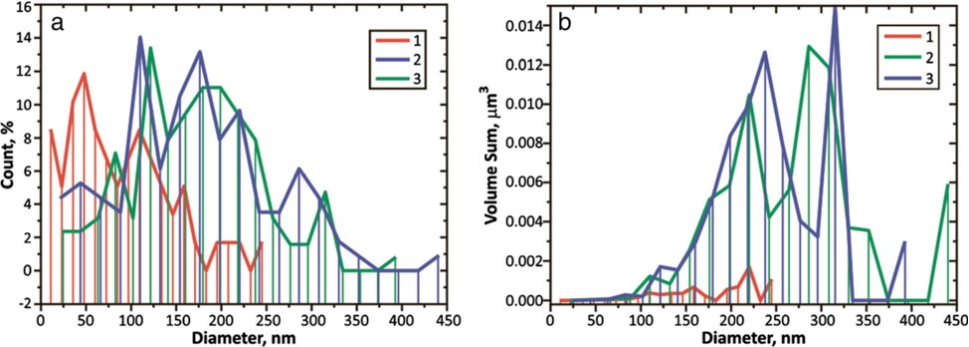
Histograms of numbers of protruding crystallites, depending on the diameter of their base. **a** Ordinary. **b** Weighted by volume. Histograms *1*, *2*, and *3* correspond to samples 9, 10, and 11, respectively

**Fig. 8:**
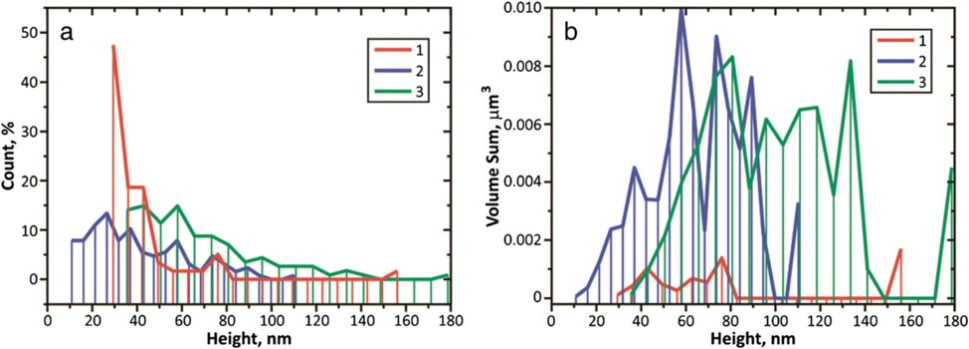
The histograms of the crystallites number, according to their height. **a** Ordinary. **b** Weighted by volume. Histograms *1*, *2*, and *3* correspond to samples 9, 10, and 11, respectively

Numbers of protruding crystallites in aluminum films of the 4th group (2D image see in Fig. 4) were also investigated by AFM. Histograms of counting of the quantity of grains, protruding from the base, with regard to the base diameter of grain (conventional and weighted by volume) are shown in Figs. 7 and 8. We should note that the volume occupied by the protruding grains increases as the deposition rate increases and constitutes from 1 to 2 % for sample 9, 11 % for sample 10, and 14 % for sample 11. The results of counting of crystallites distribution, according to their height, are shown in Fig. 8.

We should note that in the study area (5 × 5 μm^2^, Fig. 4), the maximum diameter of protruding crystallites constitutes less than 450 nm and the maximum height of the protruding crystallites is 180 nm.

### XRD of Al Films

**Fig. 9:**
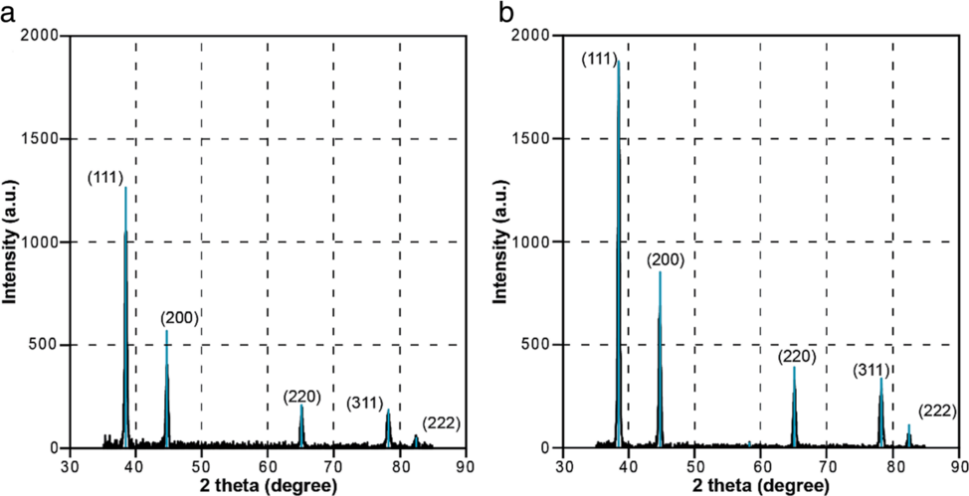
XRD patterns of the aluminum films. **a** 230 nm. **b** 340 nm

Figure 9 shows the XRD pattern of the aluminum films deposited by DC magnetron sputtering on glass substrate with a Nb adhesive layer.

The analysis confirms the polycrystalline structure of aluminum films without preferred orientation of crystallites (Fig. 9). Using Scherrer equation, the size of crystallites was estimated. The average crystal size was found to be 19 and 21 nm for Al film thickness of 230 and 340 nm, accordingly. It is in good agreement with the AFM. Calculated from X-ray reflectometry, the thickness of the film is 218 nm for Fig. 9a and 348 nm for Fig. 9b which coincides with the value given by the aluminum deposition conditions (230 and 340 nm) with an accuracy better than 10 %.

Thus, the presented results of AFM, SEM, and XRD revealed that the obtained aluminum films are polycrystalline with a great range of grain size.

The aluminum films consist of a base with smaller crystals and also of a number of grains of aluminum, which are protruding above the surface of the substrate. Our studies show that the increase of deposition rate as well as of aluminum film thickness enlarges the relative volume of metal in protruding above the surface crystallites in freshly deposited aluminum films.

The main factors responsible for the dependence of the grain size and surface roughness on the deposition rate are the features of the island growth mode (surface diffusion of adatoms, nucleation, and coalescence of Al clusters) and the influence of the residual gases (in particular oxygen), which can be incorporated into the film during deposition [26].

The observed increase in size and number of protruding grains (hillocks) of aluminum films at the increase of the deposition rate of the material is evidently analogous to that described in [27] grain growth for aluminum films with a thickness of 100 nm deposited by electron beam evaporation. This dependence can be explained by the set of the following reasons. The first of these ones is an increase of the number of arrivals to the substrate adatoms and their mobility. These results in the increase in the number of nuclei, formed on the substrate surface and after their coalescence, and leads to the increase of grain size. The second reason is an increase in the ratio of atoms, arriving on the substrate material, to the amount of precipitating ones on the surface of the growing crystal of atoms of residual oxygen that is likely to block the growth of grain.

Both reasons lead to the possibility of formation of a greater number of larger grain size, protruding above the base surface of the films with a uniform thickness when deposition rate increases.

#### Controlled Fabrication and Investigation of PAA Films

Anodic oxidation of aluminum films was performed in 0.3 M C_2_H_2_O_4_ in a two-electrode cell. To reach the given voltage (40 V), a constant current of approximately 50 mA/mm^2^ was applied. After that, we applied the potentiostatic mode.

**Fig. 10:**
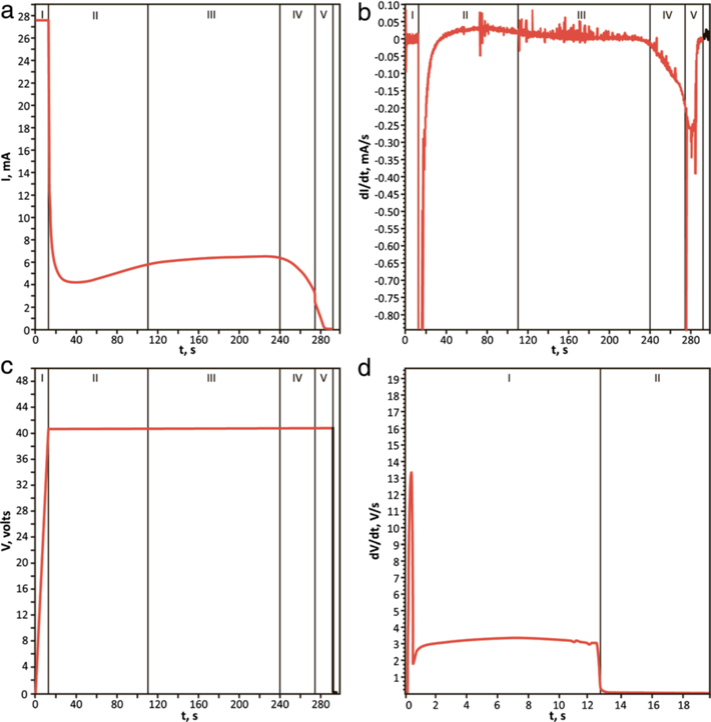
Chronometric curves, obtained by oxidation of the Al film on the glass

Chronometric curves, obtained during anodic oxidation of the aluminum film with PAA formation on the glass (Sample 4), are shown in Fig. 10.

Figure 10 illustrates the stages of PAA film formation from a thin aluminum film (230 nm), deposited on a glass with an ultra-thin (1–2 nm) adhesive layer of niobium (which appears to be oxidized by the residual gases of the vacuum chamber). The stage I corresponds to the formation of dense aluminum oxide at a constant current with an increase in voltage up to 40 V. Transition to the formation of a porous layer occurs after switching the power supply into constant voltage mode (stage II). Stage III reflects the formation of pores in the remaining aluminum film. Current density in this stage remains almost constant. Upon reaching by some parts of anodic oxide of the lower surface of the aluminum film, the current decreases due to a significant increase in resistance (stages IV and V). We attribute the sharp decrease in the current at the boundary of these stages to the loss of continuity of the residual aluminum film. However, even after that the further oxidation of aluminum islands and increase in the transparency of the PAA film on the glass occurs (stage V). At the end of anodic oxidation, the substrate visually looks completely transparent.

**Fig. 11:**
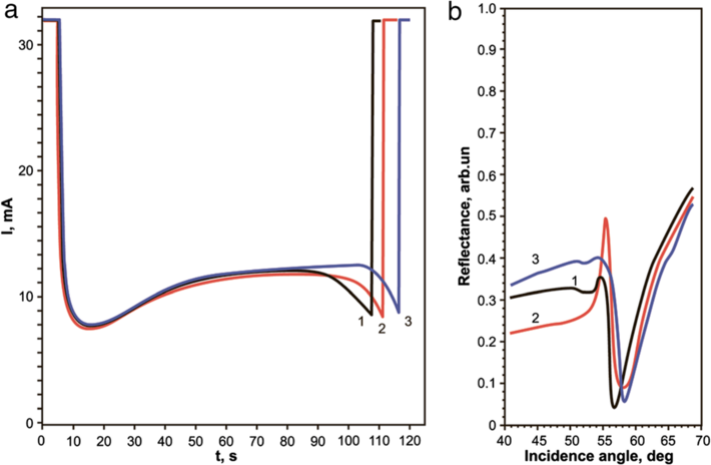
Monitoring of fabrication of MCWG PAA/Al by partial oxidation of aluminum film. **a** time-current curves, obtained during the formation of PAA/Al structures for MCWG. **b** Reflection curves of MCWG PAA/Al. Curves 1, 2, and 3 correspond to samples 9, 10, and 11, respectively

Figure 11а shows the *I*(*t*) curves, received during the formation MCWG PAA/Al by partial oxidation of samples 9, 10, and 11 (230-nm thick). Formation of PAA/Al structures by anodic oxidation was accompanied by simultaneous control of the angular dependence of the reflectance curves in the optical Kretschmann scheme using a laser with *λ* = 630 nm (Fig. 11b). The anodic oxidation was stopped on achieving of appropriate shape of reflection curve (Fig. 11b), at an average thickness of residual aluminum of 10–15 nm.

The curves *I*(*t*) for all three samples (different deposition rate of aluminum) practically coincide (Fig. 11a). Incomplete coincidence, as we believe, may be explained by slight differences in the area of anodizing masks, as well as by the fact that the thickness of the initial aluminum is somewhat variable (±5 %). The difference in the thickness of aluminum may be due to heterogeneity of the deposition on the substrate or due to time counting differences.

We should note that we never experienced the problems with delamination of the films deposited on the adhesive layer of niobium during anodic oxidation of aluminum films on glass, as it is indicated by some authors [23].

#### SEM of PAA

**Fig. 12:**
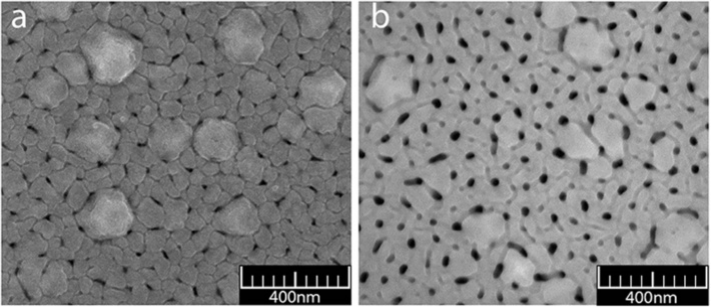
Top-view SEM micrographs of PAA. **a** After oxidation. **b** After pores widening

SEM micrographs of PAA formed through anodizing of aluminum film, which was deposited similarly to sample 7, are shown in Fig. 12. Chemical etching to widening PAA pores was being performed in 5 % H_3_PO_4_ solution at 18 °C for 30 min.

SEM images show (Fig. 12) that non-etched PAA film has irregular pores, located basically at triple points at grain boundaries. The mouth of the pores on the surface has an irregular often angular shape. The grooves at the grain boundaries of the base are clearly visible. After etching the shape of the pores at the exit to the surface acquired round shape, the grooves at the grain margins have expanded. The pore-widening rate calculated according to the change in the mean diameter of pores under etching is about 0.25 nm/min. Non-through pores of a smaller size are visible at some grains of aluminum.

The cross-section SEM image of the sample with PAA obtained by anodic oxidation from Al film published earlier by us [12] reveals parallel pores of PAA in base film which are directed perpendicular to the substrate.

**Fig. 13:**
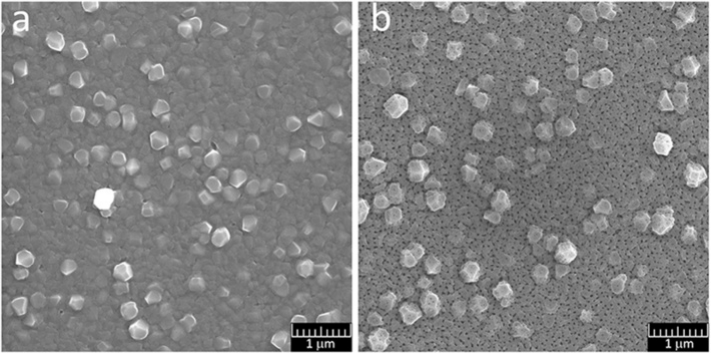
SEM image of the surface of Al film of 510-nm thick and PAA, formed from the film. **a** Al film. **b** PAA film

**Fig. 14:**
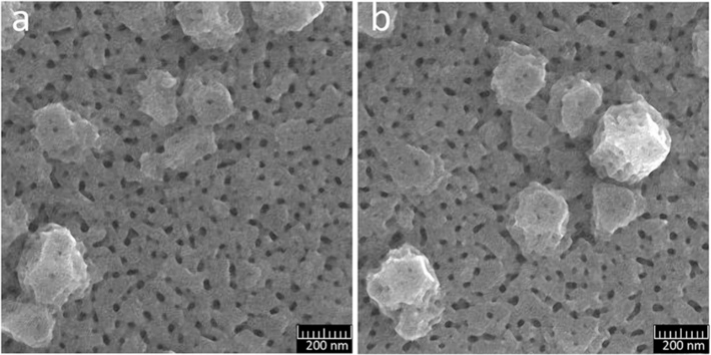
**a**, **b** Fragments of the PAA film, formed from an aluminum film with protruding crystallites

Results of SEM for films of aluminum deposited similarly to sample 8 with a thickness of 510 nm are presented in Fig. 13a and for films of porous alumina after etching (5 % H_3_PO_4_ at 18 °C for 30 min) in Fig. 13b and Fig. 14a, b. As we can see from the SEM images, pores located on a base film have a size of 25–30 nm. There is a large variation of interpore distances. Pores disposed on the protruding crystallites are smaller, their sizes are nearly 15–20 nm, and the pores are non-through. This may be explained by several reasons. Firstly, the distance from the surface grains protruding to the substrate is greater than the thickness of the base film, which was determined during the anodic oxidation. Secondly, the dynamics of pore growth on grains may differ from the pore growth on grain boundaries.

In large crystallites, protruding above the base of the aluminum film, the growth of pores is directed perpendicularly to the faces of the grains. On the faces oriented nearly perpendicularly to the substrate, there is visible tendency to their arrangement in the form of parallel rows, which is probably related to the crystallography of the grains (Fig. 14a, b). The position of these pores presumably reflects a layered structure of crystallites, observed in the SEM images of some grains of aluminum (Figs. 2 and 13).

## Conclusions

The results of SEM and AFM studies of freshly deposited aluminum films, deposited by DC magnetron, show an increase in the size and number of large crystallites with an increasing film thickness. Comparison of films of the same thickness that are deposited at different rates shows that the films deposited at higher rate also have a greater number of protruding crystallites. Increase in the deposition rate as well as in the aluminum film thickness leads to the increase in the relative volume of metal in the protruding above the surface crystallites in the aluminum films.

During anodic oxidation at 40 V in a 0.3 M aqueous solution of oxalic acid of aluminum films of 200–600-nm thickness, deposited on a glass substrate, the formation of porous anodic oxide aluminum layer with a disordered network of pores was observed. The formation of two types of pores with different sizes was revealed. The first type of pores is formed on the grain boundaries of aluminum films. These pores have large spread of sizes and distances between them. The largest pores are formed in the “triple points” between the neighboring grains. The second type of pores is then formed directly on the grains of aluminum. In large crystallites, protruding from the base of the aluminum film, the growth of pores is directed perpendicularly to the faces of the grains. The size of this type of pores is smaller than of those formed in the grain boundaries, and the dispersion of sizes in each facet is insignificant. There is a clear tendency to their ordering but the small size of grains of aluminum (100–400 nm) on the investigated films does not allow us to evaluate the degree of this ordering. The distance between pores and the degree of their ordering on the single-crystal grains is probably related to the crystallography of the grains.

## Abbreviations

AFM: atomic force microscopy
MCWG: metal-clad waveguides
PAA: porous anodic alumina
SEM: scanning electron microscopy
UTAMs: ultra-thin anodic aluminum oxide masks
XRD: X-ray diffraction
